# Impact of kinesin Eg5 inhibition by 3,4-dihydropyrimidin-2(1H)-one derivatives on various breast cancer cell features

**DOI:** 10.1186/s12885-015-1274-1

**Published:** 2015-04-14

**Authors:** Bruna C Guido, Luciana M Ramos, Diego O Nolasco, Catharine C Nobrega, Bárbara YG Andrade, Aline Pic-Taylor, Brenno AD Neto, José R Corrêa

**Affiliations:** 1 Department of Cell Biology, Laboratory of Electron Microscopy, University of Brasília (IB-UnB), Campus Universitário Darcy Ribeiro, Brasília, DF Brazil; 2 Laboratory of Medicinal and Technological Chemistry, University of Brasília (IQ-UnB), Brasília, Distrito Federal Brazil; 3 Research Laboratory of Electronics, Massachusetts Institute of Technology (MIT), Cambridge, Massachusetts USA; 4 Physics Course, Catholic University of Brasília, Brasília, Distrito Federal Brazil; 5 Laboratory of Embryology and Developmental Biology, Genetics and Morphology Department, University of Brasília (IB-UnB), Brasília, Distrito Federal Brazil

**Keywords:** 3,4-dihydropyrimidin-2(1H)-one (or thione), Breast cancer, Kinesin Eg5, Angiogenesis inhibitors, Cancer stem cells

## Abstract

**Background:**

Breast cancer is a complex heterogeneous disease and is one of the leading causes of death among women. In addressing the need for treatments of this life-threatening illness, we studied 3,4-dihydropyrimidin-2(1H)-one (or thione) derivatives (DHPMs), a class of inhibitor molecules of the Eg5 motor spindle protein that shows pronounced antitumor activity against several cancer cell lines.

**Methods:**

An *in vitro* screening was performed for identification of DHPMs with potent antitumor effects on MCF-7 and MDA-MB-231 cells and the selected DHPMs were evaluated for their inhibitory activity on Eg5 both *in silico*, using Molecular dynamics, and *in vitro* Eg5 inhibition assays. Analysis of cell death induction, proliferation, cell cycle and cancer stem cells (CSC) profile were performed by flow cytometry to assess the influence of the selected DPHMs on these important tumor features. Finally, the effects of DHPM treatment on tube formation were evaluated *in vitro* using HUVEC cells, and *in vivo* using a model on chorioallantoic membrane (CAM) of fertilized eggs.

**Results:**

We identified five DHPMs with pronounced inhibitory activity on Eg5 motor protein interfering with the proper mitotic spindle assembly during cell division. These compounds impair the correct conclusion of cell cycle of the breast cancer cells and showed to be selective for tumor cells. Moreover, DHPMs modulate the CD44^+^/CD24^−^ phenotype leading to a decrease in the CSC population in MDA-MB-231 cells, an important effect since CSC are resistant to many conventional cancer therapies and play a pivotal role in tumor initiation and maintenance. This observation was confirmed by the results which demonstrated that DHPM treated cells had impaired proliferation and were unable to sustain angiogenesis events. Finally, the DHMP treated cells were induced to apoptosis, which is one of the most pursued goals in drug development.

**Conclusions:**

The results of our study strongly suggest that DHPMs inhibit important tumorigenic features of breast cancer cells leading them to death by apoptosis. These findings firmly point to DHPM molecular architecture as a promising alternative against breast cancer.

**Electronic supplementary material:**

The online version of this article (doi:10.1186/s12885-015-1274-1) contains supplementary material, which is available to authorized users.

## Background

Breast cancer remains the most common malignancy among women and the leading cause of death, accounting for 14% of the total estimated deaths attributed to cancer in women [1,2]. In order to face this life-threatening illness, the search for new molecules capable of targeting mitosis without disrupting microtubule dynamics has significantly increased to be applied in new antineoplastic therapies [3-5]. A promising alternative to eliminate cancer cell mitotic progression relies on interference in the function of some essential mitosis molecules such as microtubule-associated proteins, particularly spindle motor proteins [6,7].

Kinesin Eg5 is one fundamental spindle motor protein and its specific role during mitosis in the assembly and maintenance of the bipolar spindle has rendered it an attractive therapeutic target that could prevent cell cycle progression through mitosis and promote tumor growth regression [8-10]. Inhibition of Kinesin Eg5 stops centrosome migration to the polar region resulting in a monoastral spindle formation [11,12], and this abnormal phenotype plays a critical role in activation of the mitotic spindle assembly checkpoint (SAC). The SAC promotes mitotic arrest at metaphase/anaphase transition through the maintenance of cdc2/cyclin B activity [8,11,13].

In addition to proliferative events that maintain tumor development, some cancer cells also have the ability to invade and colonize restricted areas belonging to other tissue types essentially through a process observed in malignant tumors named metastasis [14]. Invasion and metastasis are landmark angiogenesis-dependent events that transform a locally growing tumor into a systemic, metastatic and severe life-threatening disease [15,16].

Cancer Stem Cells (CSC), also known as initiating cancer cells, play a key role in the emergence of typical neoplastic hallmarks. These cells are able to dictate invasion, metastasis, heterogeneity, and therapeutic resistance in tumors [17,18]. Furthermore, CSC are capable of self-renewal and differentiation, and as such play a pivotal role in tumor initiation and maintenance together with spreading cancer cells to regional lymph nodes and later to other tissues and organs [19].

Resistance of putative CSC against many conventional cancer therapies is associated with two main characteristics: 1) a slow cell division rate; and 2) the ability to efflux antitumor drugs [20]. Furthermore, CSC undertake an important role in the relapse of patients post-treatment and could therefore be responsible for the incurable nature of many advanced solid tumors including metastatic breast cancer [21]. The aforementioned CSC features make these cells potential targets for cancer treatment by specific compounds that act by modulating mesenchymal-epithelial transition.

In recent years, compounds that specifically inhibit Eg5 function have been identified, such as 3,4-dihydropyrimidin-2(1H)-one (or thione) (DHPMs) [22-24]. These molecules comprise a class of heterocyclic compounds obtained through Biginelli reaction that has monastrol as their prototype [25]. The Eg5 inhibition by DHPMs is considered an attractive approach to cancer treatment since mitotic kinesins are exclusively involved in the formation and function of the mitotic spindle, and some of them are only expressed in proliferating cells [26]. The Eg5 inhibitors therefore not interfere with other microtubule-dependent processes [27,28], which are the main reason for the neurotoxicity of anti-microtubule agents [29]. It was shown that monastrol has antitumor activity against diverse cancer cell types such as renal, breast and glioma cell lines [24,30].

Here, we provide the results of a study based on the activity of thirty-seven DHPM derivatives, recently described [31] by our group, on breast cancer cells. In addition, we identified and functionally characterized the compounds that provide potent DHPM-dependent Kinesin Eg5 inhibition, and were capable of impairing metaphase/anaphase transition together with cell proliferation. Moreover, the DHPMs induce CSC differentiation into epithelial phenotype, which can controls essential properties to establishment, progression and recrudescence of tumors. Finally, the DHPM derivatives were also capable of successfully inducing cancer cells to death by apoptosis.

## Methods

### Materials and equipment

A detailed description is available in Additional file 1.

### Cells and cell culture

MCF-7 and MDA-MB-231 cell lines were used as model of breast cancer cells in this work. Our choice was based mainly on the fact that breast cancer is the most prevalent type of cancer in women worldwide and also because of the differences on metastasis capability and cancer stem cells population in these cell lines. These features affect the cell cycle and mitosis rate, which are directed linked with the predicted DHPMs effect.

MCF-7 cell line and primary culture of connective tissue cells were provided by the Laboratory of Morphology, University of Brasília (Brasília, BR). MDA-MB-231 cells were purchased from Rio de Janeiro Cell Bank (RJCB - Rio de Janeiro, BR). Cell line characterization and authentication was conducted by RJCB using short tandem repeat profiling. Primary culture cells were taken from healthy human dental pulp predominantly consisting of fibroblasts and were used as normal control cells. MCF-7 cells and fibroblasts were grown in complete DMEM and maintained at 37°C with 5% CO_2_. MDA-MB-231 cells were grown in Leibovitz L15 medium at 37°C without CO_2_. Culture media were supplemented with 10% fetal bovine serum and 25 μg/mL of gentamicin.

### Catalytic synthesis of the 3,4-dihydropyrimidin-2(1H)-one (or thione) derivatives (DHPMs)

The thirty-seven compounds tested in this work (Table 1) were provided by the Laboratory of Medicinal and Technological Chemistry, University of Brasília, and synthesized by the catalytic Biginelli reaction as previously described [31].

**Table 1:** **Synthesized DHPM derivatives**


Ent	Reagent				Prod.	Yield (%)
	R^1^	R^2^	R^3^	X		
1	Ph	Me	Me	O	**4c**	99
2	Ph	OCH_2_CH_3_	Me	S	**4d**	93
3	Ph	Me	Me	S	**4e**	83
4	4-Cl-Ph	OCH_2_CH_3_	Me	O	**4f**	87
5	4-Cl-Ph	OCH_2_CH_3_	Me	S	**4h**	80
6	4*-*Cl-Ph	Me	Me	S	**4i**	77
7	3*-*OH-Ph	OCH_2_CH_3_	Me	O	**4j**	98
8	3-OH-Ph	Me	Me	O	**4k**	80
9^a^	3-OH-Ph	OCH_2_CH_3_	Me	S	**Mon**	93
10	3-OH-Ph	Me	Me	S	**4m**	88
11	2-OH-Ph	OCH_2_CH_3_	Me	O	**4n**	84
12	2-OH-Ph	Me	Me	O	**4o**	80
13	2-OH-Ph	OCH_2_CH_3_	Me	S	**4p**	84
14	2-OH-Ph	Me	Me	S	**4q**	90
15	3-NO_2_-Ph	OCH_2_CH_3_	Me	O	**4r**	96
16	3-NO_2_-Ph	OCH_2_CH_3_	Me	S	**4t**	86
17	3-NO_2_-Ph	Me	Me	S	**4u**	86
18	2-NO_2_-Ph	OCH_2_CH_3_	Me	O	**4v**	70
19	2-NO_2_-Ph	Me	Me	O	**4x**	60
20	2-NO_2_-Ph	Me	Me	S	**4z**	60^e^
21	4-OH-3-MeO-Ph	OCH_2_CH_3_	Me	O	**4ba**	98
22	4-OH-3-MeO-Ph	OCH_2_CH_3_	Me	S	**4bc**	85
23	4-OH-3-MeO-Ph	Me	Me	S	**4bd**	85
24	H	OCH_2_CH_3_	Me	O	**4be**	96
25	Me	Me	Me	O	**4bj**	66
26	Me	OCH_2_CH_3_	Me	S	**4bk**	70
27		OCH_2_CH_3_	Me	O	**4bm**	87
28		Me	Me	O	**4bn**	79
29^b^		OCH_2_CH_3_	Me	S	**4bo**	70
30		Me	Me	S	**4bp**	71
31	3-OH-Ph			O	**4bq**	60
32^c^	3-OH-Ph			S	**4br**	72
33	3-OH-Ph			O	**4bs**	70
34^d^	3-OH-Ph			S	**4bt**	70
35		OCH_2_CH_3_	Me	O	**4bu**	66^f^
36		Me	Me	S	**4bv**	50^f^
37		OCH_2_CH_3_	Me	S	**4bx**	42^f^
38		Me	Me	O	**4by**	83

^a^Monastrol. ^b^Piperastrol. ^c^Enastron. ^d^Dimethylenastron. ^e^12 h of reaction. ^f^Product formation was above 90%, but there was considerable loss during purification column chromatography.

37 DHPM derivatives and monastrol were studied in this work.

### Cell viability assays

Fibroblasts, MCF-7 (both 3 × 10^3^/well) and MDA-MB-231 (5 × 10^3^/well) cells were plated in 96-well plates and treated with DHPMs for 24, 48 and 72 h. Cytotoxicity was determined using 3-(4,5-dimethylthiazol-2-yl)-2,5-diphenylterazolium bromide (MTT) according to the manufacturer’s instructions. Absorbance readings were measured by a spectrophotometer. Cell viability was normalized to control (vehicle only).

### Molecular dynamics

Molecular dynamics simulations (MD) of the Eg5 protein and Eg5 protein complexes with each of the five tested compounds were conducted in an aqueous environment, using the Single Point Charge – SPC – water model [32]. Analyses were performed using the GROMACS 4 computer package [33]. The dynamics utilized the 3-D protein model, collected from PDB (PDB-ID 1X88), as the initial structure. The ensembles were immersed in approximately 65,600 water molecules in dodecahedral boxes with a minimum distance of 0.7 nm between complex-box frontiers. Sodium ions were also inserted in the ensembles in order to neutralize system charges. (See details in the Additional file 1).

### Kinesin inhibition assay

Kinesin inhibition assays were performed using Kinesin ELIPA (Enzyme Linked Inorganic Phosphate Assay). Reactions were conducted in 96-well plates according to the manufacturer’s recommendations (Kinesin ELIPA Biochem Kit – BK060). The half maximal inhibitory concentration of each compound was added to the reactions (4 m - 197.3 μM; 4bt (known as dimethylenastron) - 126.9 μM; 4p - 87.54 μM; 4bc - 234.9 μM; 4x - 276.5 μM and monastrol – 110.4 μM) with readings taken at 30-second intervals for 30 minutes at room temperature using a spectrophotometer at 360 nm.

### α-tubulin immunostaining

Aliquots of 7 × 10^4^ MCF-7 cells were seeded onto 12 mm round glass coverslips placed in the base of each well of a 24-well plate. After adhesion, cells were treated for 24 and 48 h with the pre-selected compounds at their maximum non-cytotoxic concentrations to normal cells or incubated with culture medium only for the same duration (negative control). Cells were washed with PBS, fixed with 3.7% formaldehyde, permeabilized with 0.1% Triton X-100 and blocked in PBS supplemented with 1% skimmed milk, 2.5% bovine serum albumin (BSA) and 8% fetal bovine serum (FBS) at room temperature. Cells were incubated overnight with mouse anti-α-tubulin antibody (1:500) at 4°C, followed by incubation with a secondary antibody: Alexa Fluor 488 rabbit anti-mouse IgG (1:400), for 1 hour at 37°C. Nuclei were stained with 300 nM DAPI. The coverslips were mounted with *ProLong Gold Antifade* and specimens observed under a laser scanning confocal microscope.

### Transmission electron microscopy analysis

Aliquots of 8 × 10^5^ MCF-7 cells were seeded in 12-well plates and ultra-structural analysis performed on controls or after 48 h of treatment with 4p (0.4 mM). Cells were washed twice with PBS and fixed overnight with glutaraldehyde (2.5%) at 4°C. Cells were subsequently washed with 0.1 M sodium cacodylate buffer (pH 7.2) and post-fixed in 1% osmium tetroxide and 0.8% potassium ferricyanide (10 mM CaCl_2_ in 0.2 M sodium cacodylate buffer). Samples were washed twice with 0.1 M sodium cacodylate buffer (pH 7.2) and in-block staining was performed for 16 h with 0.5% uranyl acetate at 4°C. Cells were dehydrated in a graded acetone series (50-100%) and embedded in Spurr resin. Ultrathin sections were observed in a Jeol® 1011 transmission electron microscope (TEM) at 80 kV.

### Flow cytometry analysis

MCF-7 and MDA-MB-231 cells were seeded (1 × 10^5^) in 12-well plates and treated with the five pre-selected DHPMs for the determined time for each experiment. Treatment for apoptosis assay, cancer stem cell, and cell cycle analysis was conducted using 4 m (1 mM), 4bt (dimethylenastron, 0.8 mM), 4p (0.4 mM), 4bc (1.0 mM), 4x (0.8 mM) and monastrol (positive control, 1.0 mM). For proliferation assays, cells were treated with IC_50_ concentrations of each compound. Adherent and floating cells were harvested at the same tube and pelleted by centrifugation at 300 g for 5 minutes and stained. Data acquisition of these two fractions put together was performed on a FACSCalibur flow cytometer using CellQuest software and analysed using the FloJo Software.

#### Apoptosis and necrosis assay

Untreated control cells and DHPMs-treated for 72 h cell samples were stained with Annexin-V-FITC or Annexin-V-Alexa Fluor® 680 and propidium iodide according to the manufacturer’s instructions.

#### CD44^+^/CD24^−^ expression analysis

Expression level of CD44 and CD24 in treated and control MCF-7 or MDA-MB-231 cells was measured after 24 h of treatment. Cells were washed in PBS with 1% BSA. Antibodies against CD44-FITC and CD24-PE were added at the dilution suggested by the manufacturer in PBS/1% BSA and incubated on ice for 30 minutes.

#### Proliferation assay

MCF-7 and MDA-MB-231 cells were labeled with 5-(and 6-)-carboxyfluorescein diacetate succinimidyl ester (CFSE) prior to culture. After adhesion, cells were treated with DHPMs or maintained in culture medium only (control) for 72 h. The percentage of proliferative cells was calculated based on the CFSE fluorescence profile analysis of the tested samples compared to that of the fixed undivided control cells (treated with 10 μM of colchicine) using the FlowJo software.

#### Cell cycle analysis

Control and DHPM-treated cells were harvested at 24, 48 and 72 h, resuspended in ice-cold PBS and fixed with 70% ethanol on ice. Cells were then washed with PBS, harvested and incubated with propidium iodide solution (0.1% Triton X-100, 10 μg/mL propidium iodide, 100 μg/mL DNase free RNase) for 10 min at 37°C. For analysis of cell populations in each cell cycle phase, the sub-G1 picks were excluded and the plots were generated by cell cycle platform data analysis by the FlowJo Software.

### HUVEC tube formation assay

The anti-angiogenic potential of DHPMs was tested using an *in vitro* Angiogenesis Assay Kit according to the manufacturer’s instructions. Aliquots of 8 × 10^3^ HUVEC cells were resuspended in medium supplemented with endothelial cell growth supplement (1 DMEM:1 RPMI, 2% SFB, 25 μg/ml gentamicin, 15 μg/ml ECGS) with or without 30 μM, IC_50_ or 300 μM of DHPM treatment and were seeded onto the surface of the polymerized ECMatrix™ in 96-well plates. These plates were incubated for 11 hours at 37°C, in 5% CO_2_. Wells were subsequently photographed using an inverted light microscope at 20x magnification and tube formation analyzed. Seven fields per group of 3 independent experiments were quantified by pattern recognition according to manufacturer’s instructions.

### Chorioallantoic membrane (CAM) assay

DHPM anti-angiogenic potential was also tested *in vivo* using a CAM assay performed according to Ribatti, [34] with some experimental time adaptations according to Sun *et al.* [35]. A minimum of 3 fertilized eggs was used per group. On the 10th day, 1 × 10^5^ MCF-7 or MDA-MB-231 cells resuspended in supplemented culture medium (experimental control) or in medium supplemented with 500 ng of compounds 4 m, 4bt (dimethylenastron), 4p, 4bc, 4x, monastrol and genistein (as the anti-angiogenic control) were implanted into a chorioallantoic membrane (CAM) in a 2 mm^2^ hydrolysate collagen hemostatic sponge. Blood vessels were quantified on the 17th day by Wimasis Image Analysis. All blood vessels emerging from the grafts were included in the quantification.

### Statistical analysis

The quantitative results are presented as the mean ± SEM for at least three repeated individual experiments for each group. Statistical analyses were performed using GraphPad Prism 5 Software. Statistical significance of differences was determined by ANOVA with post-hoc comparison by the Bonferroni test. A P value of <0.05 was considered statistically significant.

## Results

### DHPM derivatives produce a dose and time dependent cytotoxicity and are selective for tumor cells

**Figure 1:**
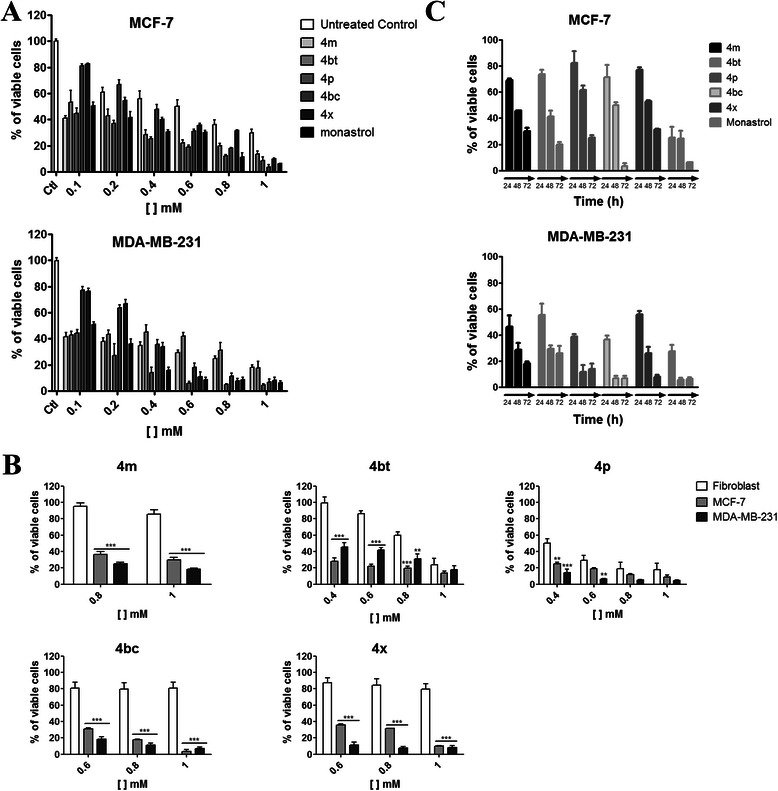
DHPM derivatives produce a dose and time dependent cytotoxicity and are selective for tumor cells. **A**, MCF-7 and MDA-MB-231 cells were treated for 72 h with 4 m, 4bt (dimethylenastron), 4p, 4bc and 4x (100 μM – 1 mM) and cell viability evaluated by the MTT assay. Columns, mean of viable cells; bars, SEM; ***(P < 0.001). **B**, Fibroblasts were treated with the compounds and concentrations that showed significant activities in breast tumor cells at 72 h and the results compared with those from MCF-7 and MDA-MB-231 cells. Columns, mean of viable cells; bars, SEM; **P < 0.01 and ***P < 0.001 compared with treated fibroblasts. **C**, MCF-7 and MDA-MB-231 cells were treated with 4 m, 4bt (dimethylenastron), 4p, 4bc and 4x at the maximum non-cytotoxic concentration to normal cells for 24 h, 48 h and 72 h and cell viability evaluated in function of time by the MTT assay. Columns, mean of viable cells; bars, SEM. **A**, **B**, **C**, Data represent the mean ± SEM of three independent experiments in triplicates.

Monastrol (a DHPM derivative) was the first known small cell-permeable molecule with potential inhibitory properties against the mitotic machinery [11]. Our work is based on compounds with the same monastrol scaffold, that are, 37 newly synthesized 3, 4-dihydropyrimidin-2(1H)-one (or thione) derivatives (see structures in Table 1). Preview screen tests were conducted in order to identify high antitumor activity against human breast tumor cells together with fewer side effects on normal cells. After 72 h of treatment, 10 of 37 compounds induced ≥ 60% of MCF-7 cells to death and 30 demonstrated the same activity on MDA-MB-231 cells (Additional file 2: Figure S1). Five of the DHPMs: 4 m, 4bt (dimethylenastron), 4p, 4bc and 4x showed the highest activities leading to a significant decrease in cell viability in both cell lines at all concentrations tested (100 μM – 1.00 mM) (Figure 1A).

To establish the optimal treatment concentration for each one of these five compounds, a cell viability assay with human fibroblasts (normal cells) was conducted using concentrations that induce at least 70% of death in breast tumor cells in both cell lines at 72 h of treatment. The highest concentration of each derivative that did not show any significant cytotoxic effects on normal cells (maximum non-cytotoxic concentration to normal cells) was selected to conduct further experiments (Figure 1B and Table 2). This experiment shows that with exception of the 4p, compounds have selectivity for tumor cells as they are in general, dose-dependent for tumor cells, but not for normal cells.

**Table 2:** **IC**_**50**_ **and maximum non-cytotoxic concentration to normal cells established for the five pre-selected compounds (4 m, 4bt (dimethylenastron), 4p, 4bc and 4x) and monastrol**

Compound	IC_50_(μM)		Maximum non-cytotoxic concentration to normal cells (mM)
	MCF-7	MDA-MB-231	
**4 m**	197.3	77.08	1.0
**4bt**	126.9	17.91	0.8
**4p**	87.5	68.38	0.4
**4bc**	234.9	249.5	1.0
**4x**	276.5	237.4	0.8
**Monastrol**	110.4	113.9	1.0

These concentrations were determined based on cells treatment for 72 h.

The half maximal inhibitory concentration (IC_50_) was calculated based on the treatment of tumor cells for 72 h (Table 2). The IC_50_ values obtained from MDA-MB-231 cells treated with 4 m, 4bt (dimethylenastron) and 4p derivatives were smaller than those achieved from monastrol. Although 4bc and 4x show higher concentrations associated to IC_50,_ their activities are not dose dependent for normal cells as they were for tumor cells (Figure 1B). Finally, the observed activity of these five selected molecules shows time-dependent effects with the best time for treatment defined as 72 h (Figure 1C).

### DHPMs induce severe morphological alterations in breast tumor cells

**Figure 2:**
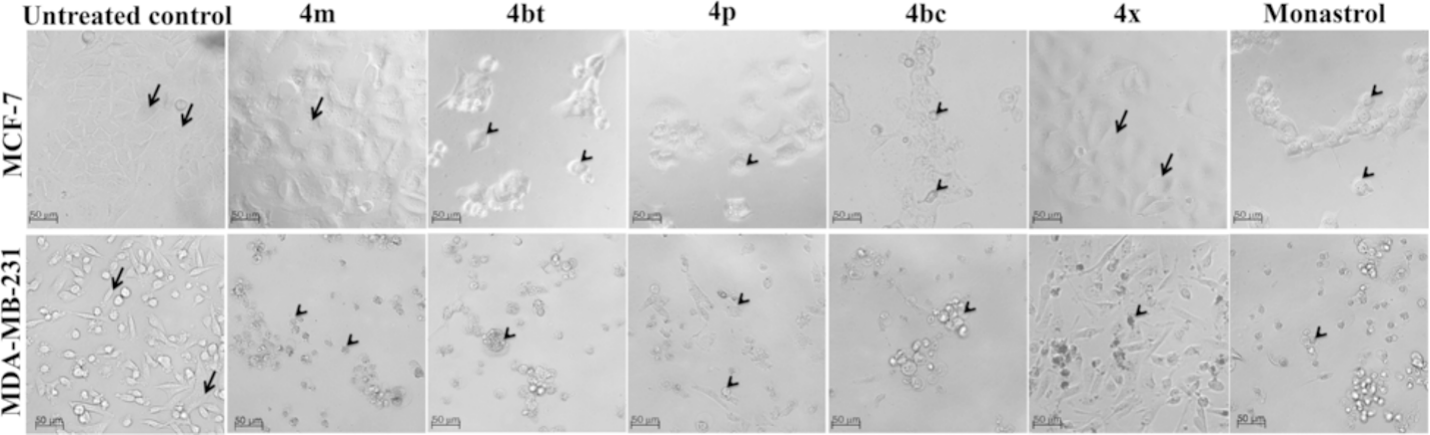
DHPM derivatives cause severe morphological alterations on breast cancer cells. MCF-7 and MDA-MB-231 cells were treated with the maximum non-cytotoxic concentration to normal cells for 72 h and morphological alterations analyzed under an inverted light microscope. Cells with elongated morphology, adherent to the surface and normal size are shown by arrows. Cells with size reduction, absence of focal adhesion points, rounded morphology and detached from the surface where they had been seeded are shown by arrowheads.

The five selected derivatives produced significant morphological alterations to MDA-MB-231 cells after 72 h of treatment (Figure 2). Only the 4bt (dimethylenastron), 4p and 4bc derivatives produced the same results with MCF-7 cells under the same treatment conditions (Figure 2). These changes were characterized by a reduction in cell size, absence of focal adhesion points, rounded morphology and minor cell detachment from the surface where they had been seeded. A significant reduction of cell number on the cover slips from treated groups in comparison to the control group was also observed. This feature suggests that many cells died as a result of the actions of the derivatives used (Figure 2). The 4 m and 4x derivatives caused no significant alterations to MCF-7 cells, similar to the observed with untreated control samples (Figure 2).

### DHPMs influence kinesin Eg5 movements in different ways

Molecular dynamics assays were performed in order to verify the binding pattern and influence of the five DHPMs on Eg5 behavior. It is believed that the protein’s movements exert important influence over its activity [36]. Thus, the *in silico* study of induced protein stiffening, together with the *in vitro* data, may provide an interesting overview of the link between derivative impact on protein movements and its inhibitory activity.

**Figure 3:**
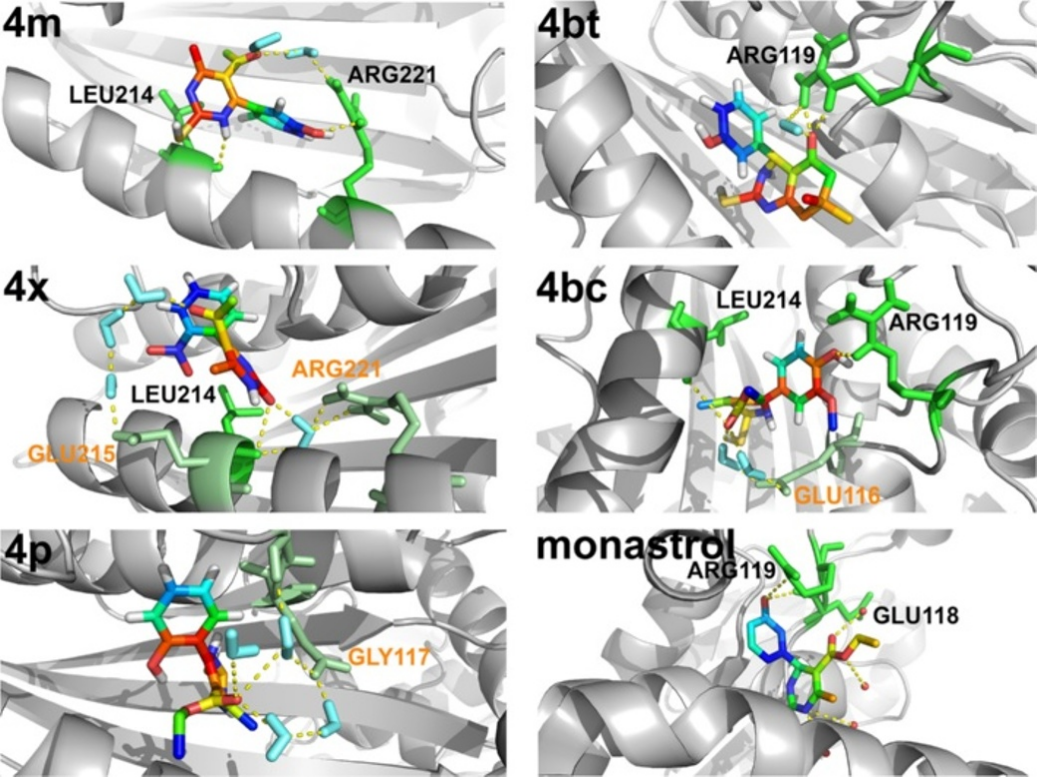
3D structure-based models of the Eg5 complexes after the MD assays. Molecular dynamics simulations (MD) of the Eg5 protein and Eg5 protein complexes with each of the five tested compounds were conducted to assess the DHMPs influence on Eg5 movements. The panels show ribbon representations of the Eg5 and Licorice representations of the tested compounds and monastrol. The yellow dotted lines show the polar contacts responsible for the maintenance of molecule linkage with the protein binding-site. The binding-region is perfectly conserved, but not the binding-residues, which lead us to realize the influence of the compound topology. Major differences may be seen in the interactions between 4 m, 4x and 4p and the Eg5 binding-site, which show completely different binding-residues. However, 4bt and 4bc retain the linkage with the Arg119 of the Eg5 when comparing to the monastrol, showing its importance in the inhibition processes. Indirect links mediated by water molecules (cyan), occur in residues (green) annotated in orange; direct links occur with residues annotated in black.

The different binding patterns observed for the DHPM derivatives tested (Figure 3) show that the interaction between some Eg5 residues and the DHPM derivative is highly significant. This is exemplified by the 4bc derivative, which interacts directly with Leu214 and Arg119, and also has two water-mediated interactions with Glu116, strongly tying the ligand-binding site (Figure 3). It is important to note that 4bc derivative interacts with Arg119, in a similar way to monastrol, but instead of interacting with Glu118, it bonds to Glu116, which ensures stability of the α-helix. The interaction with Leu214 is also noticeable as 4bc is the only derivative that binds to residues on both sides of the ligand-binding site.

Dimethylenastron (4bt) also exhibits an interesting interaction with Arg119. This interaction occurs both directly and by water-mediated means, which ensures a strong linkage between the compound and the residue. Arg119 also interacts with monastrol and 4bc, highlighting its importance during formation of the Eg5 protein ligand-binding site (Figure 3).

Derivatives 4 m and 4x interact with the Leu214 residue, but only 4 m interacts with Arg221. All of these direct interactions involve residues located at this α-helix, without interacting with residues located at the other side of the site.

Conversely, 4p does not directly interact with any residue, showing only one water-mediated link to Gly117, which shows that the related α-helix is of fundamental importance in the Eg5 catalytic site formation.

#### Principal component and RMSD analysis

Principal component analysis is a useful tool when the interest is searching for different motion modes during simulations [37]. This analysis was performed to confirm the influence of the first component during the simulation and to clarify the compounds interference on protein stability.

**Figure 4:**
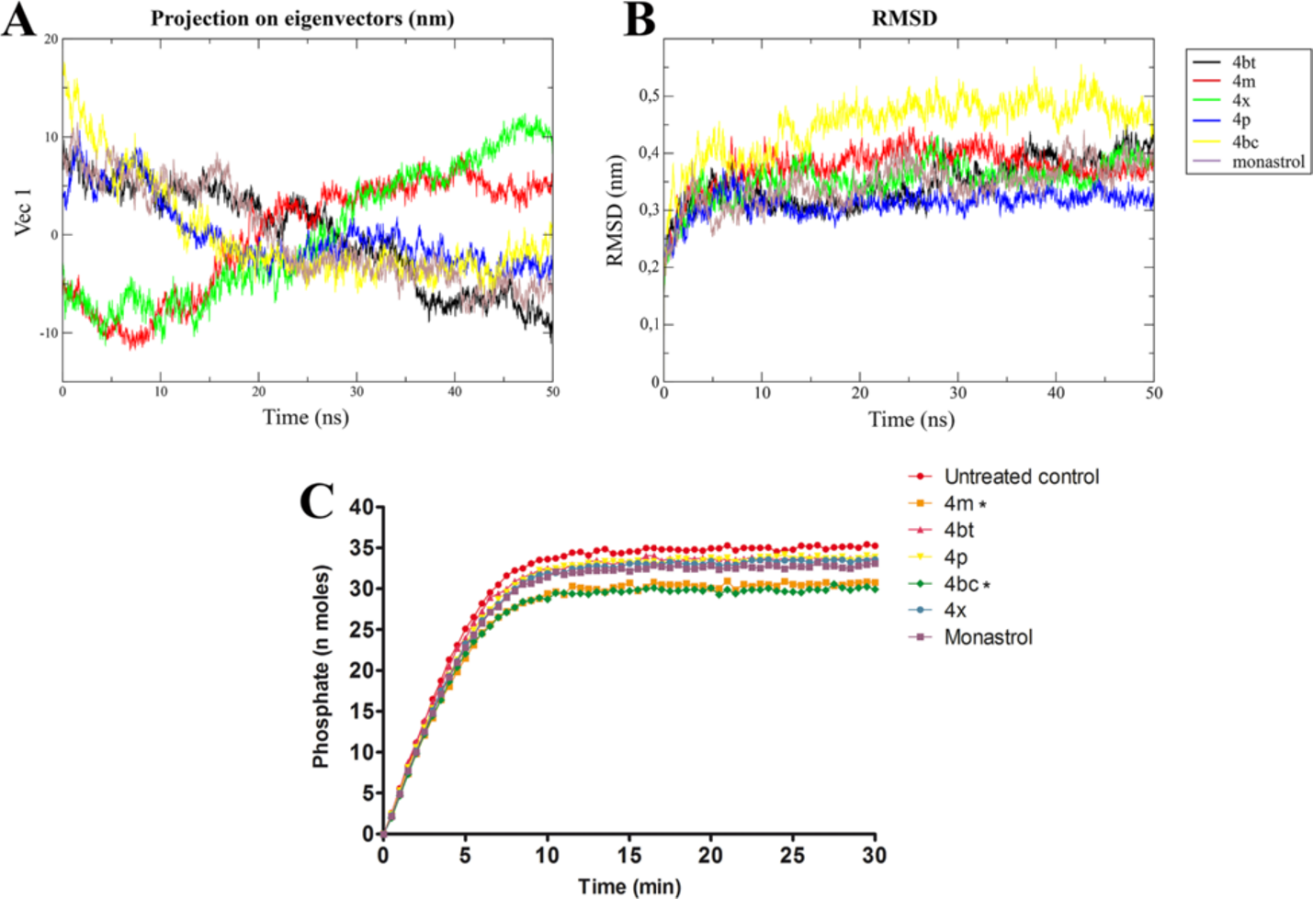
4bc leads Eg5 to a very restricted conformation and potently inhibits this protein *in vitro.***A**, Essential dynamics analysis over time was performed to assess the DHPMs interference on Eg5 protein stability. Values far from zero mean that the structure has greater movement amplitude in its first principal component. It is possible to note that compound 4bc leads the protein to a more stable conformation after the MD assays. **B**, RMSD from the initial structure over time. Compound 4bc allows the greatest initial shift compared to the other molecules, but all of them lead the protein to a stable conformation after about 15 ns. **C**, Inhibitory rate of Eg5 by DHPMs *in vitro*. Kinesin Eg5 (1 μg) was incubated with the IC_50_ of 4 m, 4bt (dimethylenastron), 4p, 4bc, 4x and monastrol with readings taken immediately after incubation at room temperature at 30 second intervals for a total reaction time of 30 minutes. Reactions were measured in spectrophotometer set in kinetic mode and an absorbance wavelength of 360 nm. Dots, mean of nmols of phosphate *versus* reaction time. *P < 0.05.

A comparison of the trajectories (Figure 4A) shows that 4bc leads the protein to a minor movement during the simulation. The 4bc derivative restricts protein movement in such a way that the first principal component gets close to zero in about 20 ns and remains (Figure 4A). Derivatives 4 m and 4x act in a similar way, but it is possible to see that 4 m leads the protein movements to a stabilized plateau after 40 ns, while 4x remains in an increasing behavior. Dimethylenastron (4bt), 4p and monastrol are in the smallest ranges of all projections, but have a final projection value further from zero than 4bc.

RMSD was also assessed to verify how different the structure becomes from the initial structural conformation during the simulation. It was noticeable that the compounds exerted influences of different magnitudes (Figure 4B): 4bc allowed an important initial shift in the protein conformation of approximately 0.5 to 20 ns, and then the structure stabilized; 4 m exerted a significant influence on the structure until approximately 10 ns, leading the protein to a shift of almost 0.4 nm; 4x, 4bt (dimethylenastron) and monastrol displayed a similar influence on protein changes, leading the RMSD to a plateau of approximately 0.35 nm; and 4p showed the least important influence, leading the structure to a plateau of 0.3 nm (Figure 4B).

### Kinesin Eg5 activity is inhibited *in vitro* by DHPM derivatives

Inhibition of kinesin Eg5 motor action by DHPMs was assessed *in vitro*. Compounds 4bc and 4 m inhibited Eg5 activity in a more pronounced way and were also more effective than monastrol. 4 m and 4bc reduced the maximum reaction rate (V_max_) from 18.67 to 16.52 and 15.30, respectively (data not shown). Compounds 4bt (dimethylenastron), 4p and 4x inhibited kinesin Eg5 in a similar way to monastrol however, this inhibitory activity was not statistically significant (Figure 4C).

### DHPMs treatment induces monoastral spindle formation and can lead human breast cancer cells to a mitotic catastrophe phenotype

**Figure 5:**
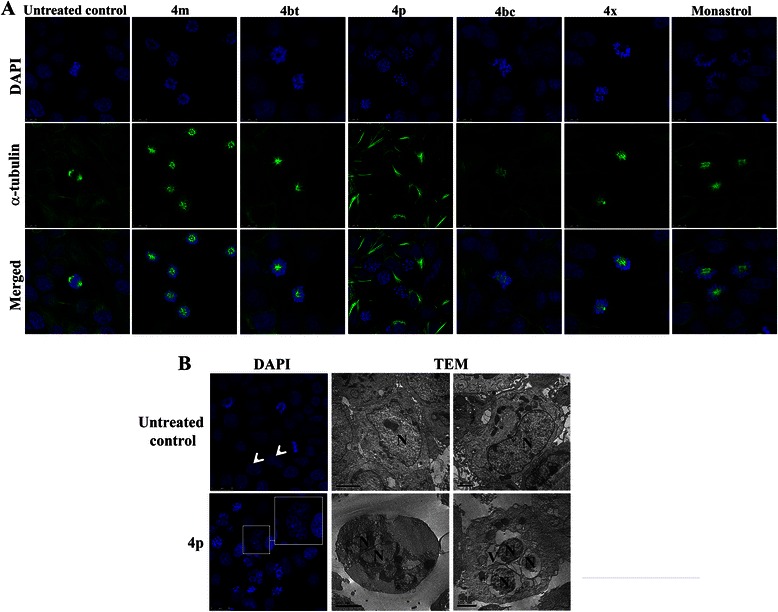
DHPMs produce monoastral spindles and 4p can lead cancer cells to a mitotic catastrophe phenotype. **A**, MCF-7 cells exposed to 4 m (1.0 mM), 4bt (dimethylenastron, 0.8 mM), 4p (0.4 mM), 4bc (1.0 mM), 4x (0.8 mM), monastrol (0.1 mM – positive control) or to culture medium only (negative control) for 24 h were immunostained with α-tubulin (green) and DNA stained with DAPI (blue). Cells were analyzed under a laser scanning confocal microscope. **B**, MCF-7 cells control or treated with 4p (0.4 mM) for 24 h had their nucleus stained with DAPI (left panels) or were processed and analyzed by Transmission Electron Microscopy after exposure to 4p (0.4 mM) for 48 h (right panels). Treated cells (lower panels) showed nuclear fragmentation with multiple micronuclei formation (MN), perinuclear vacuolization (V) and maintenance of cell membrane integrity (black arrows) compared to untreated cells (upper panels) showed nuclei with normal morphology and integrity (N) and (white arrowhead).

In order to verify the DHPMs interference with microtubule organization and mitotic spindle formation, immunofluorescence was performed using anti-α-tubulin antibody. Four of the five tested compounds (4 m, dimethylenastron (4bt), 4bc and 4x) caused monoastral spindle formation in MCF-7 cells during mitosis (Figure 5A), similar to monastrol, as previously reported [38]. These data corroborate our results obtained from the *in vitro* Kinesin inhibition assay, indicating that these compounds are indeed effective inhibitors of this protein activity. The percentage of cells with monoastral spindles that accumulate in asynchronous populations of MCF-7 cells treated for 24 h and 48 h is shown in Additional file 3: Table S1.

Treatment of MCF-7 cells with 4p (0.4 mM) produced a quite distinct pattern of microtubules organization, not observed for the other compounds/derivatives. Treated cells showed disruption of the microtubules, which were disordered and appeared to be concentrated in peripheral cell areas, adjacent to the plasmatic membrane (Figure 5A). Another observed change was the nuclear fragmentation phenotype with multiple micronuclei formation (Figure 5B), a common characteristic of a mitotic catastrophe.

In order to confirm that 4p treated cells undergo mitotic catastrophe, a supplementary transmission electron microscopy (TEM) analysis of these samples was performed. Treated cells showed nuclear fragmentation evidenced both by DAPI staining and TEM (Figure 5B). Plasma membrane integrity and a perinuclear vacuolization (Figure 5B) were also observed in a small number of cells from this sample. These observations strongly indicate that 4p treatment leads cells to a mitotic catastrophe phenotype [39].

### DHPMs-induced cell death occurs mainly by apoptosis

**Figure 6:**
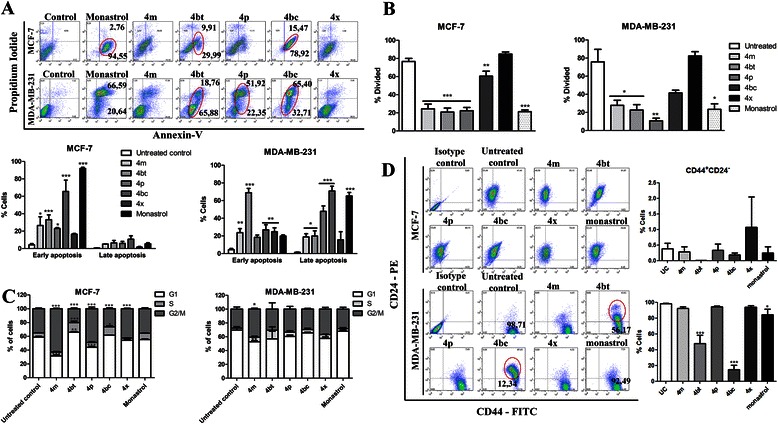
DHPMs potently repress breast tumor cell proliferation, induce apoptosis and modulate the CSC phenotype. MCF-7 and MDA-MB-231 cells control or cells treated with 4 m (1.0 mM), 4bt (dimethylenastron 0.8 mM), 4p (0.4 mM), 4bc (1.0 mM), 4x (0.8 mM) and monastrol (1 mM) were analyzed by flow cytometry to assess: the type of cell death caused by treatment for 72 h by staining cells with Annexin-V and PI **(A)**; the ability to inhibit cell proliferation after 72 h of DHPM exposure, using CellTrace™ CFSE **(B)**; the cell cycle profile after 48 h of treatment by staining the DNA content with PI **(C)**; and the CSC population after 24 h of treatment, evaluating the CD44 and CD24 expression **(D)**. Columns, mean of cells; bars, SEM, *P < 0.05; **P < 0.01; ***P < 0.001. All analyzes were performed in three independent experiments.

We subsequently determined the type of cell death induced by DHPM derivatives in breast cancer cells. The majority of cells from both cell lines died by apoptosis induced by treatment with the derivatives (Figure 6A). However, 4bc and 4bt (dimethylenastron) derivatives showed remarkable cell death induction, translated on antitumor activity. The 4bc derivative led to cell death by apoptosis: 92% and 97% of MCF-7 and MDA-MB-231 cell populations, respectively. Dimethylenastron (4bt) induced apoptosis in 39% and 83% of cell populations in MCF-7 and MDA-MB-231, respectively; thus showing similar activity to that recorded for monastrol (Figure 6A). The other compounds (4 m, 4p and 4x) also caused cell death by apoptosis, although a less pronounced manner.

These results indicate that treatment with DHPMs can foster a significant decrease in cell viability. Interestingly, the MDA-MB-231 cells are more susceptible to cell death induction by DHPM derivatives than MCF-7 cells. Minor doses and shorter treatment periods were sufficient to induce a high number of MDA-MB-231 cells to death (Figure 1A and C). Therefore, a large number of dead cells were found with a late apoptosis profile after 72 h of treatment and not in early apoptosis as for MCF-7 cells (Figure 6A).

### DHPM derivatives potently repress breast tumor cell proliferation

Proliferation is a crucial process in the maintenance and progression of cancer cells. Therefore, we monitored the effects of DHPM derivatives on proliferation of breast cancer cells, MCF-7 and MDA-MB-231, by using a CFSE assay followed by flow cytometry analysis after 72 hours of treatment.

Significant inhibition of the proliferation pattern was shown by the samples treated with 4 m, 4bt (dimethylenastron), and 4p both in MCF-7 and in MDA-MB-231 cells, as was also observed for monastrol treatment. Approximately 50% of cell proliferation was inhibited after treatment with these derivatives (Figure 6B). Derivative 4bc also showed antiproliferative activity, but at a lower level.

### Treatment with some DHPMs may result in cell cycle arrest in G2/M phase

In order to evaluate if DHPMs could impair normal cell cycle progression, the influence of the compounds on MCF-7 and MDA-MB-231 cells treated for 24, 48 and 72 h were analyzed. Our results demonstrated that after 48 h treatment, compounds 4 m, 4p and 4x caused a cell cycle arrest at G2/M in MCF-7 cells. The derivatives with high cytotoxic activity, 4bt (dimethylenastron) and 4bc, led to a decrease of cells in G2/M and an increase of cells in S phase (Figure 6C, left panel). Regarding MDA-MB-231 cells, only the 4 m derivative was able to cause cell arrest in G2/M. When considered in conjunction, these results indicate that this arrest in G2/M could not be observed in the treatments with more cytotoxic compounds because of the rapid cell elimination. No other significant change was observed in any stage of the cell cycle compared to the untreated control (Figure 6C, right panel). Results after 24 h and 72 h of treatment were less significant. Only the 4 m caused cell cycle arrest in G2/M in MCF-7 cells after 24 and 72 h of treatment and no alterations occurred in MDA-MB231 treated cells in these times (Additional file 4: Figure S2).

### DHPMs lead to a decrease in the breast CSC subpopulation

In breast cancer, a subset of markers, including: CD44^hi^/CD24^lo^, aldehyde dehydrogenase, Hoechst dye efflux, and the retention of the PKH26 lipophilic dye, have been shown to enrich CSC in various cell lines [40-42]. To investigate whether the DHPMs could influence the phenotypic profile on breast cancer cell subpopulations, CD44 and CD24 surface markers were evaluated in MCF-7 and MDA-MB-231 cells treated with each of the five derivatives.

The MCF-7 cells have a very small population of cancer stem cells with an average of 0.38% for CD44^+^/CD24^−^ cells. The treatment with DHPM has no impact on the expression of these markers and therefore does not change the amount of CSC in the MCF-7 cell population (Figure 6D and Additional file 5: Table S2). However, the results were extremely interesting regarding the influence of DHPM in modulating the expression of these molecules in MDA-MB-231 cells. Derivatives 4bt (dimethylenastron) and 4bc significantly decreased the CD44^+^/CD24^−^ subpopulation from 98.45% (untreated control) to approximately 47.83% and 14.84%, respectively (Figure 6D and Additional file 6: Table S3). We also observed a significant increase in the CD44^+^/CD24^+^ phenotype in samples treated with these derivatives. These results indicate that 4bt (dimethylenastron) and 4bc compounds might be inducing a transition of MDA-MB-231 cells to a more epithelial profile.

### DHPM derivatives inhibit angiogenesis both *in vitro* and *in vivo*

**Figure 7:**
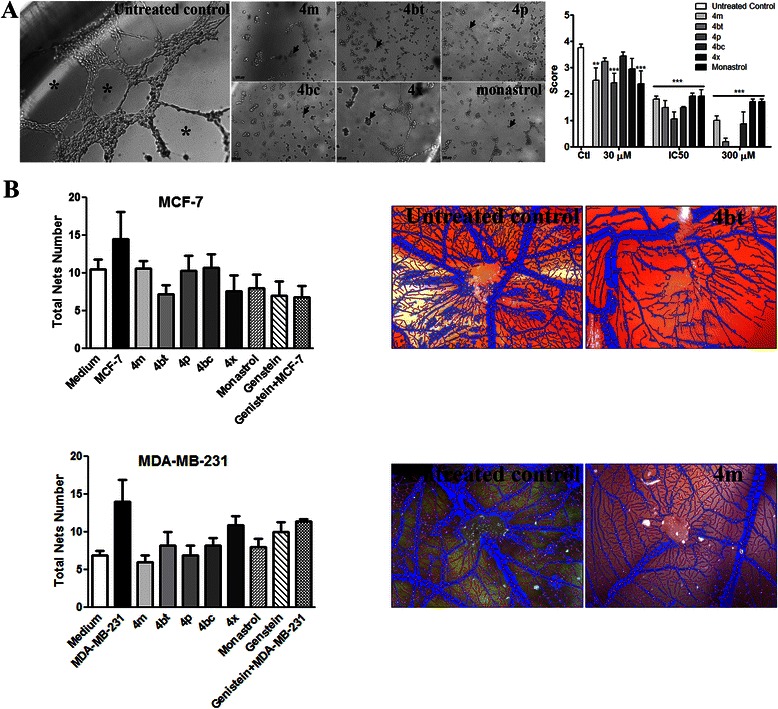
DHPM derivatives inhibit angiogenesis both *in vitro* and *in vivo.***A**, Tube formation analysis by HUVEC cells seeded onto the surface of the polymerized ECMatrix™ in 96-well plates after 11 h of treatment with 4 m, 4bt (dimethylenastron), 4p, 4bc, 4x and monastrol. Representative images of cells treated with 300 μM of each compound documented using an inverted light microscope at 20x magnification (left panel). Tube-like structures formed by HUVEC cells control (asterisks) or cells exposed to 30 μM, IC_50_ or 300 μM of 4 m, 4bt (dimethylenastron), 4p, 4bc, 4x and monastrol for 11 h, which have lost their ability to cluster in tube-like structures (arrows), were quantified by pattern recognition (right panel). Columns, means of scored tube formation; bars, SEM; **P < 0.01; ***P < 0.001 compared with untreated control. Seven frames per group of three independent experiments (n = 1) were quantified. **B**, Chorioallantoic membrane (CAM) tumor assay. MCF-7 and MDA-MB-231 cells control or treated with 500 ng of 4 m, 4bt (dimethylenastron), 4p, 4bc, 4x, monastrol or genistein (antiangiogenic control) were implanted into CAM and on the 17th day of development the quantification of total nets was performed by Wimasis Image Analysis (left panel). Columns, mean of total nets (n = 3–5); bars, SEM. A representative image of the most effective treatment for each cell compared with the respective controls can be observed (right panels).

The effects of DHPM treatment on tube formation were evaluated *in vitro* using HUVEC cells, and *in vivo* using a model on chorioallantoic membrane (CAM) of fertilized chicken eggs. The *in vitro* assay demonstrated that HUVEC cells were capable of building tubes by connecting to neighboring cells forming a rich meshwork of branching capillary-like tubules (Figure 7A). However, following DHPM treatment at different concentrations (30 μM, IC_50_ and 300 μM), the intercellular connection was abrogated and HUVEC cells failed to form tubes. It was observed at the higher concentration that the capillary-like tubes were interrupted and most cells presented spherical morphology, either in isolation or aggregated in small clumps (Figure 7A). In addition, all the compounds resulted in a significant decrease in HUVEC tube formation mainly at the higher concentration (300 μM) as quantified by patter recognition (Figure 7A right panel). Derivatives 4p and 4bt (dimethylenastron) were associated with the more pronounced anti-angiogenic morphological aspect, which suggests that these derivative properties substantially interfered with the ability of HUVEC to form capillary-like structures *in vitro*.

To assess whether DHPM derivatives could also inhibit vessel formation by breast tumor cells, an *in vivo* model based on CAM of fertilized chicken eggs was performed. These results indicated that all derivatives were capable of subtly inhibiting new blood vessel formation at very low doses (500 ng) (Figure 7B). Although the anti-angiogenic properties presented by DHPM on the CAM assay were not statistically significant, our data suggests a marked reduction in vessel convergence toward tumor cell implantation and vessel thickness (Figure 7B - right panel). It is probable that the reduction in the total number of networks formed by vessels would be much more exacerbated at higher concentrations.

## Discussion

In this study, we conducted an *in vitro* examination of the different aspects of cytotoxicity induced by 3,4-dihydropyrimidin-2(1H)-one (or thione) derivatives in MCF-7 and MDA-MB-231 cells. Several results suggest that DHPMs can inhibit kinesin Eg5 in a specific manner, leading to a critical error in mitotic spindle assembly, which in turn induces cell death by apoptosis. Apoptosis induction of cancer cells is the most pursued goal in drug development, as recently reviewed [43], due to its natural clearance effect, no association with inflammatory mechanisms, and its related role in tissue regeneration [44].

Cell proliferation is a crucial process relating to cancer initiation, maintenance and progression [45]. In our studies, cell treatments with low concentrations of 4 m, 4bt (dimethylenastron), and 4p derivatives act primarily by preventing cell proliferation in both cell lines. These data suggest a very interesting DHPM cytostatic effect which is a useful property in targeting cancer cells that are resistant to apoptosis induction and have a poor response to proapoptotic agents.

Another very interesting finding was that at much lower concentrations, compound 4p showed high cytotoxic activity on breast tumor cells, however this effect was also well pronounced on normal cells which is probably due to the fact that this compound binds to another target rather than the Eg5 motor protein. Despite its lack of direct interaction with kinesin Eg5, as demonstrated by MD analysis (Figure 3) and visualized by α-tubulin immunostaining (Figure 5A), 4p was able to induce cell death by mitotic catastrophe (Figure 5B).

According to Vitale and coworkers (2011), failing mitoses are often associated with chromosomal breakages and deficient karyokinesis, which lead to gross nuclear alterations (micronucleation and multinucleation) that constitute the most prominent morphological traits of mitotic catastrophe [46,47]. In addition, other typical features of this event are mitosis blockage, mitotic spindle disorganization and chromosome segregation failure [41]. Due to the activation of several death pathways following mitotic catastrophe, the induction of this event is considered a valid mechanism to subvert drug resistance [48]. It was also shown that molecules such as monastrol, dimethylenastron (4bt), K858 and ARRY-520 are able to trigger mitotic catastrophe and cell death by inhibiting kinesin [11,49,50].

Our results indicated 4bc as a potent and specific antitumor agent. This molecule showed dose-dependent lethal action against tumor cells, but not for normal cells, and together with 4bt (dimethylenastron) is considered the most promising of the compounds tested. These results corroborate molecular dynamics data, in which reveals these compounds form more stable bonds with Eg5 protein residues, and these connections are made in the common residues that monastrol binds to [51]. The binding-site sealing, promoted by the linkage of 4bc, prominently inhibit protein mobility, causing a significant reduction of its major mobility-dependent functions. The MD results show that this mechanism of action can be the main cause of Eg5 inhibition by 4bc. Since the other compounds did not show a relevant stiffening promotion, as observed for 4bc, it is possible to assume that there is a strong relationship between protein movements and its activity. Furthermore, it is possible to understand the importance of Arg119 in catalytic binding-site formation, since the majority of inhibitory molecules interact with this residue. Reviewing the essential dynamics and RMSD results; it is easy to see that 4bc leads Eg5 structure to an interesting shift, and ultimately to an important stabilization, different from the other compounds. This stabilization in a different structural conformation may be the key to the competitive inhibition of Eg5 activity.

In addition to these properties, 4bc and 4bt (dimethylenastron) also affect a very important property of tumor establishment and maintenance - the presence of cancer stem cells. CSC are defined by their tumor-initiating properties which appear to be responsible for driving tumor growth, recurrence, and metastasis [40,52]. We detected a considerable decrease in CSC population (CD44^+^/CD24^−^) in MDA-MB-231 cells treated with 4bc and 4bt (dimethylenastron) and an increase in the CD44^+^/CD24^+^ population. This was a surprising result in that it was previously verified that CSC isolated from breast cancer cell lines are resistant to radiation and chemotherapy [53,54]. Furthermore, these treatments can select for the outgrowth of therapy-resistant cancer cell subpopulations that are more tumorigenic, invasive, and stem like [55,56]. Hence, cancer therapies may be rendered ineffective because the bulk of cancer cells within a tumor may be eliminated while leaving slow cycling cells behind, such as CSC which are also resistant to apoptosis and proceed to regenerate the tumor [57-59]. This alteration in cells that become more epithelial (CD44^+^/CD24^+^) [60] allowed them to acquire susceptibility to cytotoxic effects produced by DHPMs or other anticancer agents. Further studies are necessary in order to characterize the molecular aspects related with CSC under DHPMs derivatives treatment.

## Conclusions

Our results provide evidence of potent Eg5 inhibitors, the DHPMs derivatives, which prevent normal mitotic spindle formation during cell division inducing breast tumor cells to apoptosis, besides to show that several cellular processes are impaired by DHPMs activity differently of many works that show only viability data just from tumoral cells. Thus, the major overall findings obtained from the analyses are:

i) DHPM derivatives are potent Eg5 inhibitors and have potent activity against MCF-7 and MDA-MB-231 cells with low cytotoxicity to normal cells affording a great advantage over current drugs widely used in cancer treatment;
ii) Two compounds (4bt and 4bc) are more efficient than monastrol, a widely recognized inhibitor of Eg5 activity;
iii) The DHPMs are able to interfere, *in vitro,* with the development of essential properties for tumor establishment and progression such as cell proliferation, angiogenesis, and CSC phenotype;
iv) The cytotoxic effect of DHMPs is accompanied by the induction of apoptosis in MCF-7 and MDA-MB-231 cells
v) DHPM architecture is a promising chemotherapeutic candidate because of its selective bioactivity in tumor cells, which should be further evaluated by *in vivo* assays.

## Additional files

Additional file 1:
**Supplementary Methods – A detailed methodology about material, equipment and molecular dynamics).**

Additional file 2: Figure S1. Effects of DHPM derivatives on MCF-7 and MDA-MB-231 cell viability.
Additional file 3: Table S1. Quantification of monopolar spindles occurred as a result of treatment of MCF-7 cells with DHPM derivatives.
Additional file 4: Figure S2. Effects of DHPM derivatives on MCF-7 and MDA-MB-231 cell cycle profile after 24 and 72 h of treatment.
Additional file 5: Table S2. Percentage of subpopulations defined by the combination of stem cell markers CD44 and CD24 in MCF-7 cells.
Additional file 6: Table S3. Percentage of subpopulations defined by the combination of stem cell markers CD44 and CD24 in MDA-MB-231 cells.

## Abbreviations

BSA: Bovine serum albumin
CAM: Chorioallantoic membrane
CFSE: 5-(and 6-)-carboxyfluorescein diacetate succinimidyl ester
CSC: Cancer stem cell
DHPMs: 3,4-dihydropyrimidin-2(1H)-one (or thione)
ELIPA: Enzyme linked inorganic phosphate assay
FBS: Fetal bovine serum
HUVEC: Human umbilical vein endothelial cell
MCF-7: Michigan Cancer Foundation 7 breast cancer cell line
MD: Molecular dynamics
MDAMB231: Breast cancer cell line derived from metastatic site (pleural effusion)
MTT: 3-(4,5-dimethylthiazol-2-yl)-2,5-diphenylterazolium bromide
PDB: Protein data bank
SAC: Spindle assembly checkpoint
SPC: Single point charge
RMSD: Root-mean-square deviation
TEM: Transmission electron microscope
