# Supplementary material for: Impact of kinesin Eg5 inhibition by 3,4-dihydropyrimidin-2(1H)-one derivatives on various breast cancer cell features
Source: BMC Cancer. 2015 Apr 14;15:283. doi: 10.1186/s12885-015-1274-1 (PMC4411898; doi:10.1186/s12885-015-1274-1)
Supplement: Additional file 1: — Supplementary Methods – A detailed methodology about material, equipment and molecular dynamics). [file 12885_2015_1274_MOESM1_ESM.pdf]

Materials and Equipment

The thirty-seven compounds analyzed in this work were synthesized and fully characterized in the Laboratory of Medicinal and Technological Chemistry, University of Brasília [1].

Cell cultures were maintained using DMEM, Leibovitz – L15 and RPMI media, Fetal Bovine Serum (FBS) and 0,25% trypsin/EDTA from GIBCO – Life Technologies (Carlsbad, CA, USA).

The antibodies used: Monoclonal anti-α tublin– Clone DM 1A produced in mouse purchased from Sigma Aldrich (Louis, MO, USA); Alexa Fluor® 488 rabbit anti-mouse; CD44 Mouse Anti-Human mAb (clone MEM-85) - Fluorescein (FITC) conjugate, and CD24 - Mouse Anti-Human - (R-PE) IgG all from Invitrogen – Life Technologies (Carlsbad, CA, USA). The isotype controls used were IgG1-PE (SC-2866) and IgG2b-FITC (SC-2857) antibodies purchase from Santa Cruz Biotechnology (Santa Cruz, CA, USA).

The reagents used were: MTT (3-(4,5-Dimethylthiazol-2-yl)-2,5-Diphenyltetrazolium Bromide) from Molecular Probes (Life Technologies, Carlsbad, CA, USA); DNAse free RNAse; Annexin-V-FITC; Annexin-V-Alexa Fluor® 680; DAPI, CellTrace™ CFSE Cell Proliferation Kit and ProLong Gold Antifade from Invitrogen (Life Technologies, Carlsbad, CA, USA); Colchicine; DMSO; Triton X-100 and BSA from Sigma-Aldrich (St. Louis, MO, USA); ATP from Invivogen (San Diego, CA, USA); Kinesin ELIPA Biochem Kit from Cytoskeleton (Denver, CO, USA), Endothelial cell growth supplement (ECGS) and *In Vitro* Angiogenesis Assay Kit from Millipore (Billerica, MA, USA).

Absorbance was measured by a Spectramax M5 spectrophotometer and analysis conducted using the SoftMax Pro5.2 software, both from Molecular Devices

(Sunnyvale, California, USA). Flow cytometry was performed using a BD FACSCalibur flow cytometer (BD Biosciences, San Jose, CA, USA). The flow cytometer data analyses were conducted using the FlowJo software v.5.2.7 (Tree Star, Inc., Ashland, OR, USA). Immunofluorescence analyses were performed using a laser scanning confocal microscope (Leica Microsystems, Wetzlar, Germany). Morphological and ultrastructural analyses were performed using an Inverted Microscope (Zeiss Axiovert 100, Germany) and a Jeol® 1011 transmission electron microscope at 80 kV (Jeol Tokyo, Japan), respectively. CAM analyzes employed the Wimasis Image Analysis Software (Wimasis GmbH, Munich, Germany).

Statistical analysis was performed using the GraphPad Prism 5, GraphPad Software, Inc. (CA, USA).

Molecular Dynamics

The geometry of water molecules was constrained using the SETTLE algorithm [2]. All atom bond lengths were linked by the LINCS algorithm [3]. Electrostatic corrections were made by Particle Mesh Ewald algorithm [4], with a cut-off radius of 1.4 nm in order to minimize the computational time. The same cut-off radius was also used for van der Waals interactions. The list of neighbors of each atom was updated every 10 simulation steps of 2 fs. The conjugate gradient and steepest descent algorithms were implemented for energy minimization. The system subsequently underwent pressure (NPT ensemble) and temperature (NVT ensemble) normalization, using the stochastic dynamics integrator together with the leap-frog algorithm. After steps of energy minimization and temperature and pressure equilibration, a step of leap-frog algorithm position restraint was carried out. The compound topologies were constructed using the PRODRG server [5]. Simulations were conducted at 300 K *in*

silico, using the GROMOS96 force field. The total time for each ensemble simulation, performed in triplicate, was 50 ns.

References

1. Ramos LM, Guido BC, Nobrega CC, Corrêa JR, Silva RG, de Oliveira HCB, Gomes AF, Gozzo FC, Neto B a D: **The Biginelli reaction with an imidazolium-tagged recyclable iron catalyst: kinetics, mechanism, and antitumoral activity.** *Chemistry* 2013, **19**:4156–68.

2. Miyamoto S, Kollman PA: **Settle: An analytical version of the SHAKE and RATTLE algorithm for rigid water models**. *J Comput Chem* 1992, **13**:952–962.

3. Hess B, Bekker H, Berendsen HJC, Fraaije JGEM: **LINCS: A linear constraint solver for molecular simulations**. *J Comput Chem* 1997, **18**:1463–1472.

4. Darden T, York D, Pedersen L: **Particle mesh Ewald: An N·log(N) method for Ewald sums in large systems**. *J Chem Phys* 1993, **98**:10089.

5. Schüttelkopf AW, van Aalten DMF: **PRODRG: a tool for high-throughput crystallography of protein-ligand complexes.** *Acta Crystallogr D Biol Crystallogr* 2004, **60**(Pt 8):1355–63.
