# Supplementary material for: Impact of kinesin Eg5 inhibition by 3,4-dihydropyrimidin-2(1H)-one derivatives on various breast cancer cell features
Source: BMC Cancer. 2015 Apr 14;15:283. doi: 10.1186/s12885-015-1274-1 (PMC4411898; doi:10.1186/s12885-015-1274-1)
Supplement: Additional file 2: Figure S1. — Effects of DHPM derivatives on MCF-7 and MDA-MB-231 cell viability. [file 12885_2015_1274_MOESM2_ESM.pdf]

A

MCF-7

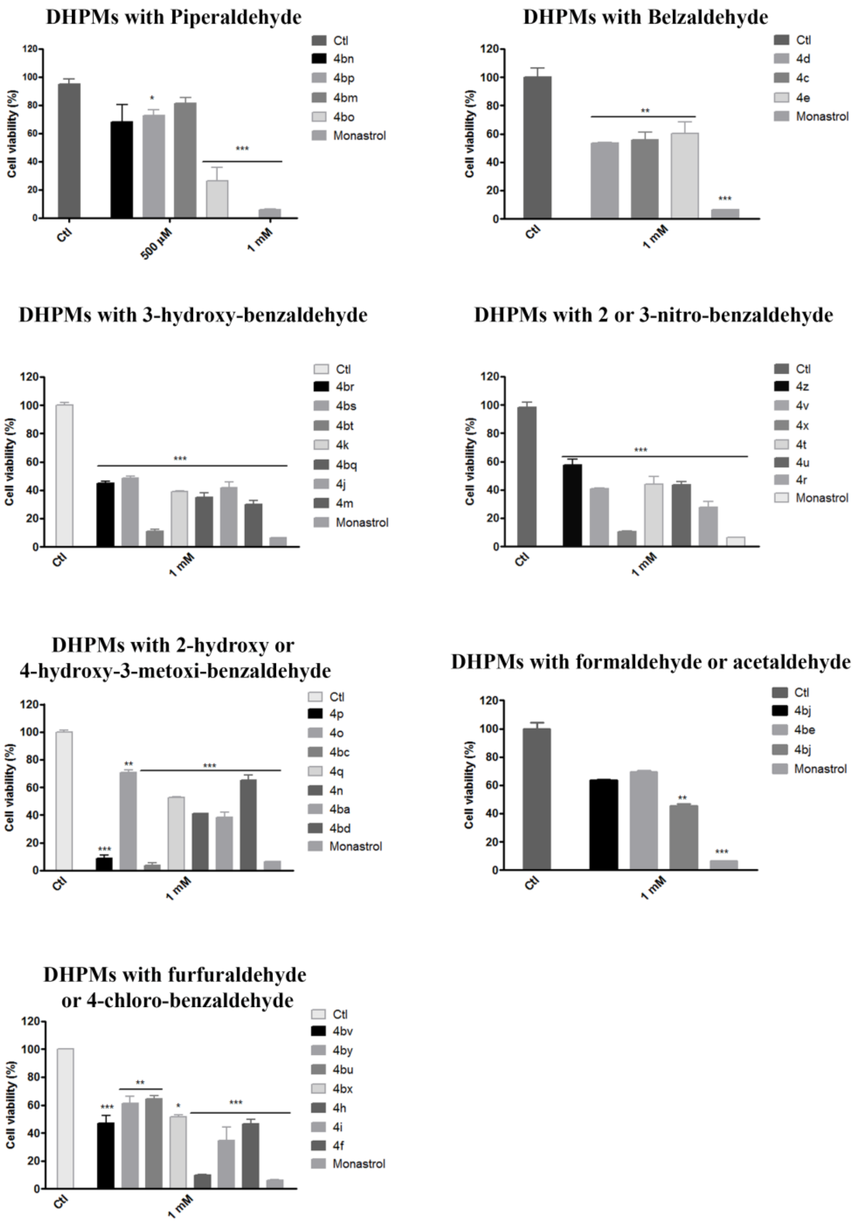
DHPMs with Piperaldehyde
Cell viability (%)
0 20 40 60 80 100 120
Ctl
500 µM
1 mM
*
***
Ctl
4bn
4bp
4bm
4bo
Monastrol
DHPMs with Belzaldehyde
Cell viability (%)
Ctl
1 mM
**
***
Ctl
4d
4c
4e
Monastrol
DHPMs with 3-hydroxy-benzaldehyde
Cell viability (%)
Ctl
1 mM
***
Ctl
4br
4bs
4bt
4k
4bq
4j
4m
Monastrol
DHPMs with 2 or 3-nitro-benzaldehyde
Cell viability (%)
Ctl
1 mM
***
Ctl
4z
4v
4x
4t
4u
4r
Monastrol
DHPMs with 2-hydroxy or
4-hydroxy-3-metoxi-benzaldehyde
Cell viability (%)
Ctl
1 mM
***
**
***
Ctl
4p
4o
4bc
4q
4n
4ba
4bd
Monastrol
DHPMs with formaldehyde or acetaldehyde
Cell viability (%)
Ctl
1 mM
**
***
Ctl
4bj
4be
4bj
Monastrol
DHPMs with furfuraldehyde
or 4-chloro-benzaldehyde
Cell viability (%)
Ctl
1 mM
***
**
*
***
Ctl
4bv
4by
4bu
4bx
4h
4i
4f
Monastrol

B **MDA-MB-231**

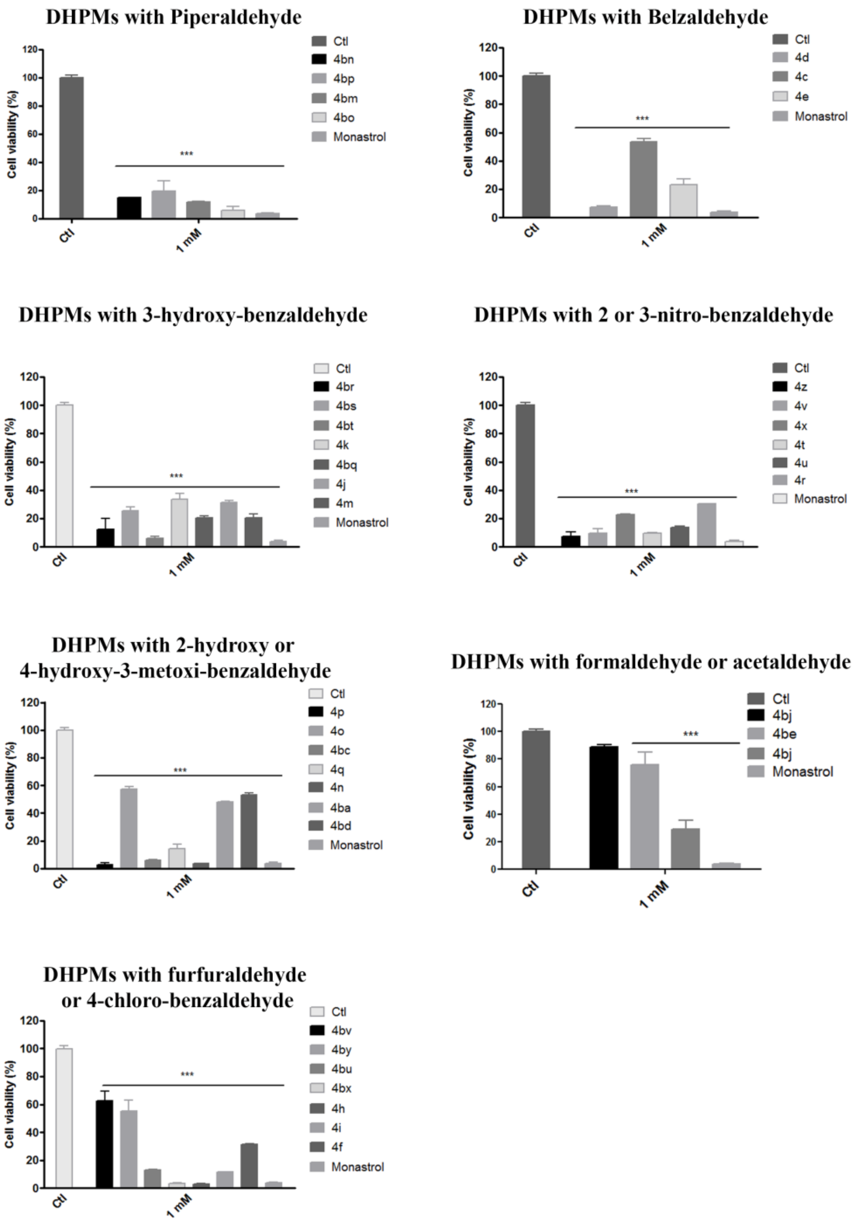

Figure S1. Effects of DHPM derivatives on MCF-7 (**A**) and MDA-MB-231 (**B**) cell viability. Breast tumor cells were treated with the thirty-seven DHPM derivatives (1.0 mM) for 72 h and cell viability determined by MTT assay. Data represent the mean ± SEM of 3 independent experiments each performed in triplicates. *P<0.05, **P<0.01 and ***P<0.001 versus untreated control group.
