# Supplementary material for: Impact of kinesin Eg5 inhibition by 3,4-dihydropyrimidin-2(1H)-one derivatives on various breast cancer cell features
Source: BMC Cancer. 2015 Apr 14;15:283. doi: 10.1186/s12885-015-1274-1 (PMC4411898; doi:10.1186/s12885-015-1274-1)
Supplement: Additional file 3: Table S1. — Quantification of monopolar spindles occurred as a result of treatment of MCF-7 cells with DHPM derivatives. [file 12885_2015_1274_MOESM3_ESM.pdf]

Table S1. Quantification of monopolar spindles occurred as a result of treatment with DHPM derivatives. MCF-7 control cells or cells treated for 24 or 48 h with 4m (1 mM), 4bt (dimethylenastron), 4p, 4bc, 4x or monastrol were labeled with anti-α-tubulin antibody. The percentage of dividing cells showing presence of monoastral and bipolar spinles formation determined (n = 300).

**Experimental Group**	**Monoastral Spindle (%)**		**Bipolar Spindle (%)**	
	**24 h**	**48 h**	**24 h**	**48 h**
Control	25.70	23	74.30	77
4m (1.0 mM)	100	100	0	0
4bt (0.8 mM)	99.35	80	0.65	20
4p (0.4 mM)	0	0	0	0
4bc (1.0 mM)	44.35	89.67	55.75	10.33
4x (0.8 mM)	50.80	87.99	49.20	12.01
Monastrol (0.1 mM)	97.66	92	2.33	8
