# Supplementary material for: Impact of kinesin Eg5 inhibition by 3,4-dihydropyrimidin-2(1H)-one derivatives on various breast cancer cell features
Source: BMC Cancer. 2015 Apr 14;15:283. doi: 10.1186/s12885-015-1274-1 (PMC4411898; doi:10.1186/s12885-015-1274-1)
Supplement: Additional file 4: Figure S2. — Effects of DHPM derivatives on MCF-7 and MDA-MB-231 cell cycle profile after 24 and 72 h of treatment. [file 12885_2015_1274_MOESM4_ESM.pdf]

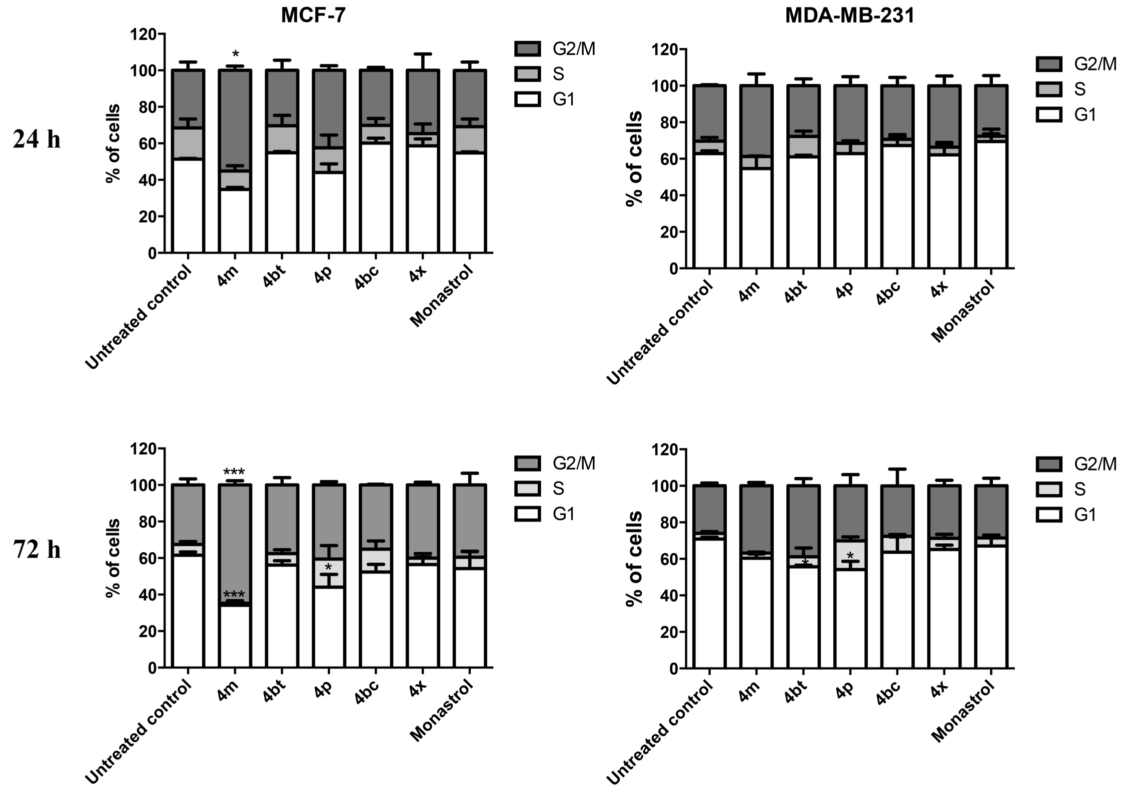

Figure S2. DHPMs can cause an arrest in G2/M in breast cancer cells. MCF-7 and MDA-MB-231 cells control or cells treated with 4m (1.0 mM), 4bt (dimethylenastron 0.8 mM), 4p (0.4 mM), 4bc (1.0 mM), 4x (0.8 mM) and monastrol (1 mM) were analyzed by flow cytometry to assess the cell cycle profile after 24 and 72 h of treatment by staining the DNA content with PI. Columns, mean of cells; bars, SEM, * $P<0.05$; *** $P<0.001$. Analyzes were performed in three independent experiments.
