# Supplementary material for: Impact of kinesin Eg5 inhibition by 3,4-dihydropyrimidin-2(1H)-one derivatives on various breast cancer cell features
Source: BMC Cancer. 2015 Apr 14;15:283. doi: 10.1186/s12885-015-1274-1 (PMC4411898; doi:10.1186/s12885-015-1274-1)
Supplement: Additional file 5: Table S2. — Percentage of subpopulations defined by the combination of stem cell markers CD44 and CD24 in MCF-7 cells. [file 12885_2015_1274_MOESM5_ESM.pdf]

Table S2. Percentage of subpopulations defined by the combination of stem cell markers CD44 and CD24 in MCF-7 control cells or treated with 4m, 4bt (dimethylenastron), 4p, 4bc, 4x or monastrol for 24 h. Data represent the mean ± SEM of 3 independent experiments.

MCF-7 Group	PHENOTYPES (MEAN ± SEM)			
	**$CD44^-/CD24^-$**	**$CD44^-/CD24^+$**	**$CD44^+/CD24^-$**	**$CD44^+/CD24^+$**
Control	2.14 ± 0.15	65.21 ± 9.23	0.38 ± 0.18	32.26 ± 9.20
4m (1.0 mM)	1.01 ± 0.12	55.59 ± 15.09	0.28 ± 0.15	43.10 ± 15.06
4bt (0.8 mM)	3.09 ± 0.46	34.92 ± 19.45	0.005 ± 0.005	61.97 ± 19.91
4p (0.4 mM)	2.83 ± 0.59	66.42 ± 10.57	0.33 ± 0.20	30.40 ± 9.78
4bc (1.0 mM)	3.90 ± 0.070	46.49 ± 18.49	0.18 ± 0.07	49.42 ± 18.49
4x (0.8 mM)	2.45 ± 0.52	61.35 ± 20.83	1.065 ± 0.97	35.11 ± 19.33
Monastrol (1.0 mM)	1.64 ± 0.43	68.56 ± 5.93	0.2400 ± 0.20	29.53 ± 5.30
