# Supplementary material for: Impact of kinesin Eg5 inhibition by 3,4-dihydropyrimidin-2(1H)-one derivatives on various breast cancer cell features
Source: BMC Cancer. 2015 Apr 14;15:283. doi: 10.1186/s12885-015-1274-1 (PMC4411898; doi:10.1186/s12885-015-1274-1)
Supplement: Additional file 6: Table S3. — Percentage of subpopulations defined by the combination of stem cell markers CD44 and CD24 in MDA-MB-231 cells. [file 12885_2015_1274_MOESM6_ESM.pdf]

Table S3. Percentage of subpopulations defined by the combination of stem cell markers CD44 and CD24 in MDA-MB-231 control cells or treated with 4m (1.0 mM), 4bt (dimethylenastron), 4p, 4bc, 4x or monastrol for 24h. Data represent the mean ± SEM of 3 independent experiments. * P<0.05. *** P<0.001 versus the untreated control.

MDA-MB-231	PHENOTYPES (MEAN ± SEM)			
Group	**$CD44^-/CD24^-$**	**$CD44^-/CD24^+$**	**$CD44^+/CD24-$**	**$CD44^+/CD24^+$**
Control	0.03 ± 0.006	0.003 ± 0.003	98.45 ± 0.47	1.507 ± 0.47
4m (1.0 mM)	0.01 ± 0.003	0.017 ± 0.012	92.57 ± 1.62	7.390 ± 1.611
4bt (0.8 mM)	0.01 ± 0.00	0.0 ± 0.0	47.83 ± 10.60 ***	52.14 ± 10.60 ***
4p (0.4 mM)	0.02 ± 0.009	0.003 ± 0.003	94.45 ± 1.13	5.507 ± 1.13
4bc (1.0 mM)	0.01 ± 0.003	0.003 ± 0.003	14.84 ± 5.27 ***	85.13 ± 5.26 ***
4x (0.8 mM)	0.01 ± 0.003	0.020 ± 0.0200	93.88 ± 2.12	6.08 ± 2.10
Monastrol (1.0 mM)	0.00 ± 0.003	0.007 ± 0.003	84.41 ± 6.97 *	15.56 ± 6.96 *
